# Targeting pericyte retention in Diabetic Retinopathy: a review

**DOI:** 10.1080/07853890.2024.2398200

**Published:** 2024-09-13

**Authors:** Forrest Bohler, Lily Bohler, Varna Taranikanti

**Affiliations:** a Department of Foundational Medical Studies, Oakland University William Beaumont School of Medicine, Rochester, MI; b College of Letters and Science, Montana State University, Bozeman, MT

**Keywords:** Diabetic retinopathy, pericyte, retinal disease, pericyte retention, treatment options

## Abstract

Diabetic retinopathy is a common yet severe complication of diabetes mellitus and is the leading cause of blindness in middle-aged adults. After years of poorly managed hyperglycemia, complications begin as non-proliferative diabetic retinopathy but can then progress into the proliferative stage marked by neovascularization of the retina. Multiple pathologic mechanisms caused by chronic hyperglycemia damage the retinal vasculature leading to pericyte drop out and the progression of the disease. This review outlines the major pathways of pathogenesis in diabetic retinopathy, highlighting the protective role pericytes play in preserving the blood-retinal barrier. Given the loss of this cell line is a defining feature of the disease, ways in which to prevent pericyte dropout within retinal vasculature is discussed, targeting various pathogenesis pathways of diabetic retinopathy.

## Introduction

Diabetes mellitus (DM) is the most common metabolic disorder in the world. It is estimated that 463 million people globally have either Type 1 or Type 2 diabetes.^1^ The underlying cause in individuals with DM is an abnormal insulin response from either a total lack of endogenous insulin production (Type 1) or a resistance to its effects (Type 2), both resulting in chronic hyperglycemia (CH). Diabetic retinopathy (DR) is a severe yet common complication of DM as it is the leading cause of blindness in adults aged 25-65. Within 2 decades of the disease, it is estimated that nearly all Type 1 diabetics develop some degree of DR, and approximately 60% of Type 2 diabetics develop the disease during this same time period.^1–3^

DR is also a disease that has a greater impact on those who come from a lower socioeconomic status (SES) as these patients are at an increased risk of poorer prognosis. Findings have shown that for Type 1 diabetics, low SES is an independent risk factor for DR and leads to the earlier development of DR compared to those with a higher SES.^4^ Other studies show that patients with DR who qualify for Medicaid, a government insurance program only offered to individuals of lower SES, have worse adherence to follow-up appointments with providers to manage the treatment of their DR. This ultimately leads to poorer DR outcomes due to lack of surveillance and treatment of the disease.^5^ While SES can have an impact on DR, DR can in turn have a negative impact on SES and the ability of a patient’s independence. Many patients report an inability to participate in routine daily activities such as mobility, driving, reading, working, and engaging in social interactions. The loss in ability to properly manage their DM is particularly concerning since this can lead to even worsened outcomes related to DM complications if the individual does not have access to personal assistance to make up for these deficits in their everyday life.^6^ DR is the most common microangiopathic complication resulting in visual impairment and blindness in both developed and developing countries.^1^

During this time period before DR develops, the best treatment options to prevent or delay the onset of DR is aggressive treatment centered around lower carbohydrate intake and increased exercise as these are effective non-pharmacological methods to lower blood sugar levels.^7^ Various medications are also available to treat CH. Insulin therapy is essential for Type 1 diabetics whose CH is due to an inability to synthesize insulin. Exogenous insulin may also be needed for Type 2 diabetics whose etiology stems from an acquired resistance to insulin. There are various oral antihyperglycemic agents available for patients after being diagnosed with diabetes that operates on a systemic level. Some of these include insulin secretagogues, biguanides, insulin sensitizers, alpha-glucosidase inhibitors, incretin mimetics, amylin antagonists, and sodium-glucose co-transporter-2 (SGLT2) inhibitors. Oral hypoglycemic drugs such as gliclazide have long since been shown to be an effective treatment option for diabetics to delay the onset and progression of DR to more severe stages by lowering fasting blood glucose levels.^8^ Exogenous insulin administration, however, has a paradoxical effect on DR and is actually associated with the worsening of disease progression.^9–11^ Although the exact mechanism is not fully understood, it is thought that the rapid improvement in diabetic control from exogenous insulin has a synergistic effect with vascular endothelial growth factor (VEGF) that is present in high amounts in the retinas of patients with DR to promote vascular proliferation.^12^

Hence, this review highlights the current understanding of the pathways that contribute to the development of DR and provides an overview of the pathogenesis of DR, the current therapeutic options, and the potential targets for future drug development.

## Disease classification

### Non-Proliferative Diabetic Retinopathy

The first stage of DR is Non-Proliferative Diabetic Retinopathy (NPDR) and usually occurs in the first 15-20 years of the disease progression of DM. Symptoms of NPDR vary for individuals. The initial stages of NPDR may be asymptomatic but can also lead to loss of visual acuity usually as a result of diabetic macular edema (DME) and macular ischemia.^13^^,^^14^

Although there are multiple types of macular edema, DME is the most common subset and is considered one of the severest complications of DR. It affects approximately 3.8% of all diabetics above the age of 40.^15^ DME develops as fluid from retinal vasculature leaks into the surrounding retinal tissue causing swelling. Swelling that occurs in the macula, which is responsible for a patient’s central vision and fine detail, often leads patients to present with blurred vision and/or color distortion.^16^

### Proliferative Diabetic Retinopathy

The final and most severe stage of DR is Proliferative Diabetic Retinopathy (PDR) which is almost always accompanied by visual disturbances. PDR is characterized by neovascularization in the retina leading to adherent vessel formation. These new blood vessels are poorly formed and prone to leakage and hemorrhage.^17^ They are created as a compensatory measure in response to decreased oxygen supply to the retina. Once an individual reaches the point of PDR, the course of the disease becomes more unpredictable as there is an increased risk of further complications such as DME and retinal detachment.^18^

### Pathogenesis overview

While the progression and development of DR is multi-factored, this review will focus on three major pathways: the Polyol Pathway, the Oxidative Stress Pathway and the Hypoxia Pathway. These three pathways contribute to cumulative damage of the endothelial cells (EC) and disrupt the fragile relationship between ECs and their supportive pericytes ultimately resulting in the loss of these pericytes. Pericyte dropout then leads to the further progression of DR and the subsequent development of the clinical symptoms seen in patients with both NPDR and PDR.^19^^,^^20^

### The Polyol Pathway

The polyol pathway is an alternate metabolic pathway that generates sorbitol from glucose utilizing aldose reductase (AR). Since AR has a high Km for glucose, the production of sorbitol is minimized under normal glycemic conditions in preference of the hexokinase pathway.^21^ Patients with CH, however, have increased levels of sorbitol production since primary glucose metabolic pathways are overwhelmed.

The damaging effects of sorbitol accumulation are two-fold. AR utilizes nicotinamide adenine dinucleotide phosphate (NADPH) as a cofactor, and increased AR activity is associated with decreased levels of NADPH.^19^ NADPH is also needed for the proper function of an enzyme with powerful antioxidant capabilities, glutathione. Impaired glutathione activity decreases the body’s ability to scavenge free radicals and leads to increased levels of oxidative stress, which can damage retinal vasculature and endothelial cells (EC).

**Figure 1.**
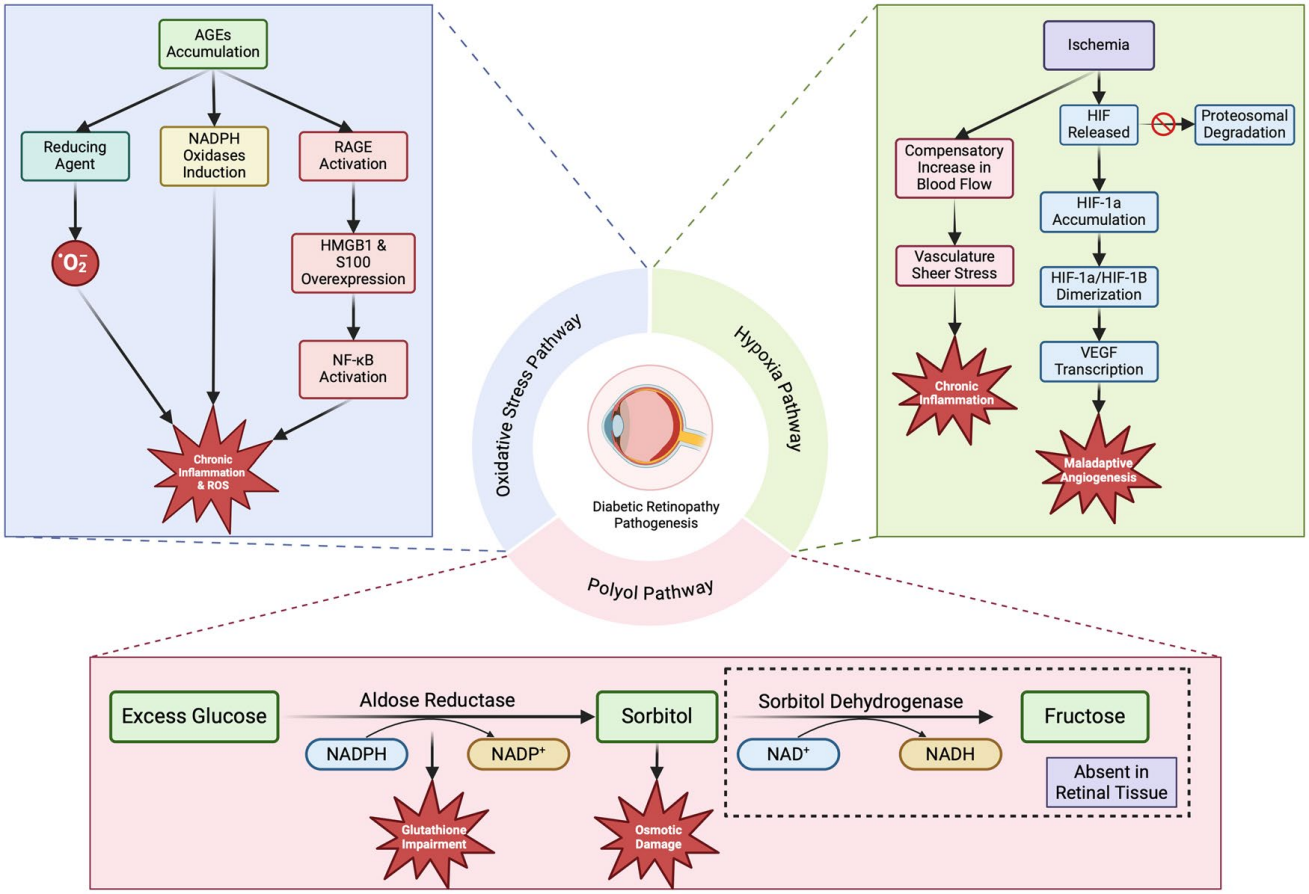
Diabetic Retinopathy Pathogenesis pathways. This figure was generated by the authors and granted a publication license with BioRender.com.

In addition to oxidative stress, sorbitol itself has a toxic effect when accumulated due to increased osmotic damage in cells.^19^ In most tissues found throughout the body, the enzyme sorbitol dehydrogenase (SDH) converts sorbitol into fructose as to avoid the toxic effects from its sorbitol accumulation. Retinal tissue, however, lacks SDH leaving it vulnerable to sorbitol accumulation (Figure 1).^22^

### The Hypoxia Pathway

Hypoxia in diabetics is a common systemic issue that has particularly damaging effects on the retina as it can lead to multiple harmful outcomes. Lack of oxygen supply to ECs can lead to inflammation and compromise the blood-retinal barrier (BRB). This chronic inflammatory state can promote ischemia due to the formation of retinal micro thrombosis that can occlude vasculature and reduce blood supply. In response to the body’s inability to provide sufficient oxygen and nutrients to the retina, a localized increase in blood flow is often seen, which can result in additional damage through increased shear stress on vessels.^20^ When increased localized blood flow is not enough to meet the oxygen demands of retinal tissue, DNA-binding proteins known as Hypoxia Inducible Factors (HIFs) are stabilized. HIFs are constitutively transcribed and translated in vascular cells, but under conditions of sufficient oxygen supply HIFs are short-lived due to proteasomal degradation. Under hypoxic conditions, however, proteasomal degradation is hindered and an HIF subtype, HIF-1a, begins to accumulate in the nucleus of cells. HIF-1a then dimerizes with another HIF subtype, HIF-1B, and binds to DNA, stimulating the transcription of target genes.^23^ One of these target genes is vascular endothelial growth factor (VEGF) which plays a fundamental role in angiogenesis leading to many of the proliferative issues seen in DR (Figure 1).^24^

### The Oxidative Stress Pathway

Damage from the oxidative stress pathway primarily occurs due to an overproduction of reactive oxygen species (ROS). Aside from the contributing effects of the Polyol pathway through impaired glutathione function, there are additional mechanisms resulting from CH that contribute to increase ROS production. One of these mechanisms involves the production of advanced glycation end products (AGEs) in hyperglycemic states. AGEs are a heterogenous class of biomolecules resulting as the product of non-enzymatic reactions of a reducing sugar with a protein, lipid, or nucleic acid.^25^ Given that AGEs are highly reactive molecules, they have the ability to donate electrons and form superoxide anions.^26^ Additionally, they have the ability to induce NADPH Oxidases, which have been shown to increase ROS production and even inhibit the body’s protective antioxidant capabilities.^27^^,^^28^

AGEs have also been implicated as a significant ligand in the activation of several inflammatory pathways such as the receptor for AGEs (RAGE). In patients suffering from PDR, activating ligands in the RAGE pathway such as AGEs, high mobility group box 1 (HMGB1) protein, and S100 protein are overexpressed.^29–31^ Following RAGE activation, nuclear factor kappa-B (NF-κB) is then activated, triggering a cascade of events that promote vascular dysfunction, inflammation, and oxidative stress.^32–34^ Additionally, this AGEs-associated inflammatory response has been implicated in the damage of cell lines in the retina’s neurovascular units such as glial cells leading to further damaging downstream effects.^35^ Moreover, increased levels of AGEs have been shown to induce VEGF expression in retinal tissue, further contributing to the formation of abnormal and adherent blood vessels seen in PDR that predispose patients to macular edema (Figure 1).^36^^,^^37^

### Pericyte in retinal health

Pericytes are present in vasculature throughout the body, but their concentration can vary. In areas that require a high degree of barrier function to prevent fluid leakage such as the BRB, the pericyte to EC ratio is as high as 1:1 underscoring their importance in maintaining a healthy retina.^20^ Pericytes wrap around ECs and provide a supportive role to these cells. Under healthy conditions, they can also prevent the unnecessary growth of ECs that can lead to the production of unstable retinal vasculature. In patients with DR, the loss of pericytes has been observed which suggests their absence plays a role in the poorly formed neovasculature that arises in PDR which can lead to visual disturbances.^38^ Additionally, ischemic conditions in the retina can lead to contraction of pericytes which further affects blood flow to downstream tissue yet the mechanism behind this contraction is unclear and continues to be debated.^39^^,^^40^

### Endothelial cell damage and pericyte dropout

**Figure 2.**
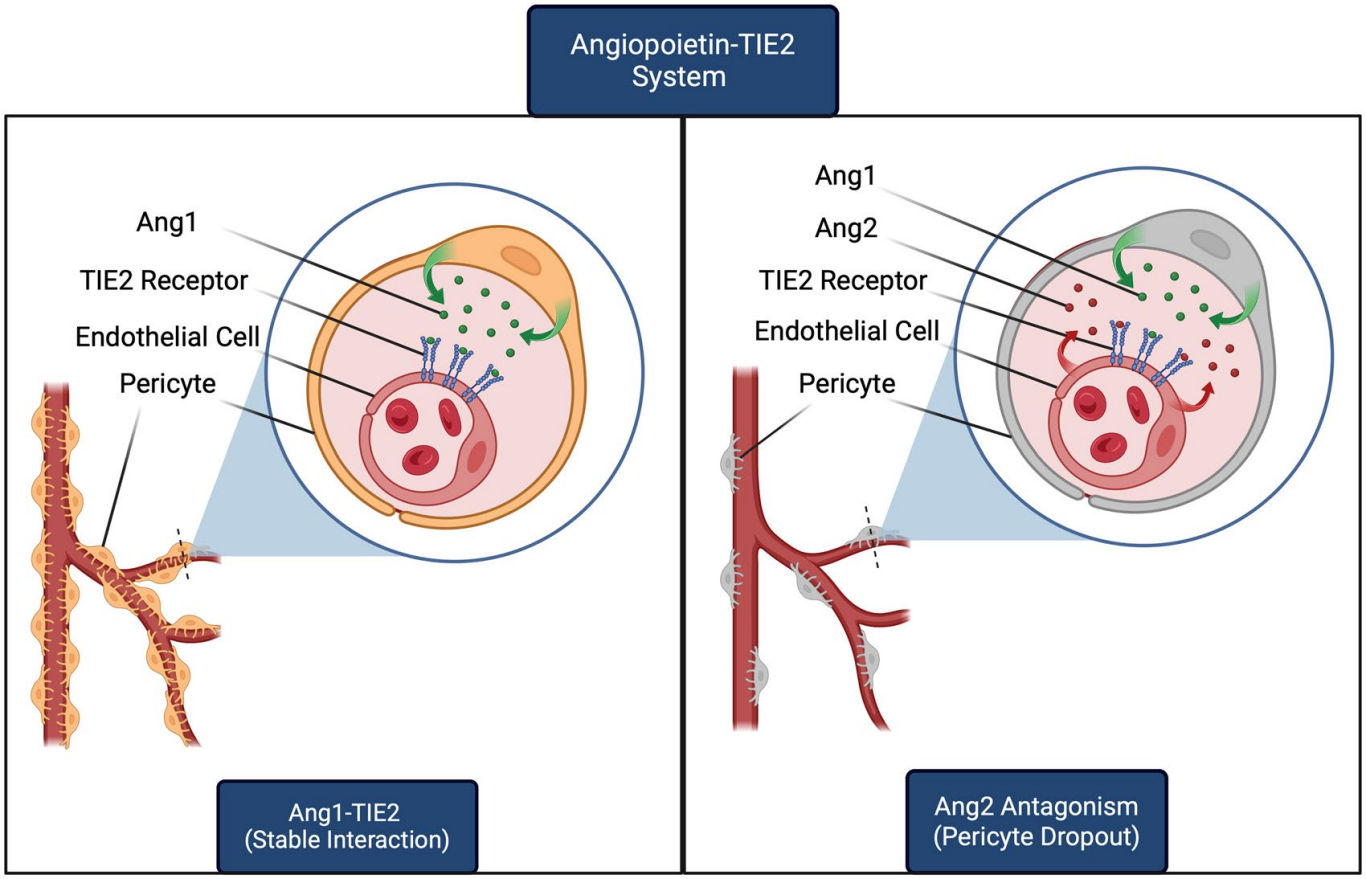
Angiopoietin-TIE2 interaction between pericytes and endothelial cells retinal vasculature. This figure was generated by the authors and granted a publication license with BioRender.com.

The maintenance of the BRB is vital for the proper functioning of the retina. The two key players in the BRB are retinal ECs that make up the inner lining of the vessels and the pericytes that cover the ECs and are crucial in stabilizing new vasculature. The loss of pericytes around ECs is common in DR and is attributed to the damaging effects of CH.^38^ It is believed that the angiopoietin-TIE2 system is largely responsible for the normal interaction between ECs and pericytes to stabilize vasculature. In non-pathological states, angiopoietin-1 (Ang-1) is generated by pericytes and stimulates TIE2 signaling in ECs, which is responsible for stabilizing the interaction between ECs and pericytes (Figure 2). The damage that occurs through the various mechanisms in DR promotes inflammation of the ECs (Figure 1). As a result, these ECs produce angiopoietin-2 (Ang-2), which is an antagonist of the Ang-1-TIE2 signaling pathway (Figure 2). The disruption of this pathway destabilizes the EC-pericyte relationship, which promotes pericyte dropout.^41^

One early microvascular finding that can be seen in the preliminary stages of DR is a “bridging” phenomenon of cells spanning the inter-capillary space, connecting two or more proximal vessels. Findings demonstrate that these bridges increase significantly in retinal microvasculature of both human retina samples and in mouse models with poor hyperglycemic control.^42^ These cells are thought to be of pericyte origin as they express a unique combination of biomarkers specific to pericytes: neuron-glial antigen 2 (NG2), platelet-derived growth factor-beta (PDGF-β), and absence of platelet and endothelial cell adhesion molecule 1 (PECAM-1).^42^^,^^43^ Additionally, these cells expressed connexin 43, a key component of intercellular gap junction complexes, indicating that these bridges are likely involved in communication between capillaries.^42^^,^^44^ These bridges also express nestin and filamentous actin suggesting they likely play a role in mechanical stability of damaged vessels.^42^^,^^45^^,^^46^ Building off the presumption that these intervascular bridges are derived from pericytes, others have theorized that these bridges are the results of pericyte detachment and migration away from healthy microvasculature, contributing to cascade of negative downstream events that occur with the loss of pericytes residing in the perivascular space.^47^ This hypothesis is further supported in findings in mouse models that reveal these bridges express Myh11, a contractile protein unique to pericytes.^47^

The subsequent loss of pericytes after bridge formation compromises the integrity of these mature vessels and leads to the formation of microaneurysms, increased vascular permeability, and hard exudates seen in NPDR. The absence of pericytes also lays the groundwork for progression into PDR by sensitizing ECs to VEGF. The production of VEGF then promotes the formation of new blood vessel growth and is the fundamental protein involved in angiogenesis.^38^

### Current treatment options

Upon diagnosis of NPDR, clinicians tend to focus on both systemic management of a patient’s CH and direct treatment of ophthalmic manifestations. Systemic treatment is centered around hyperglycemia, hypertension, and hyperlipidemia management.^48^ The most common ocular-specific treatment option in the proliferative and non-proliferative phase of DR is to target angiogenesis.^48^^,^^49^ Anti-VEGF agents have become the standard of care to treat DME and is used frequently in the management of PDR. Bevacizumab, ranibizumab, and aflibercept are the primary intravitreal injections used by retina specialists. Anti-VEGF treatments act in one of three ways: blocking gene expression of VEGF, inactivating VEGF directly, or blocking VEGF receptors.^50^ One of the most common anti-VEGF medications used is bevacizumab. This drug was originally designed to target angiogenesis in cancer patients, but its utility in combating retinal neovascularization was soon discovered. As a humanized monoclonal antibody, bevacizumab works by binding directly to the VEGF protein, eliminating VEGF’s ability to interact with VEGF receptors and exert its angiogenic effects.^51^ Aflibercept is another common anti-VEGF drug that is advantageous as has a high binding affinity for VEGF. Rather than utilizing monoclonal antibodies, aflibercept is a recombinant fusion protein, consisting of a human IgG molecule with human VEGF receptor proteins on the IgG’s FC-region. Once administered, the FC-region of aflibercept is able to tightly bind circulating VEGF and prevent it from interacting with endogenous receptors. While anti-VEGF intravitreal injections are highly beneficial to many patients, they are not without inherent risks. Ocular hemorrhage, transient acute elevations in intraocular pressure, and rhegmatogenous retinal detachments have all been noted as potential adverse side effects of this treatment.^52^

More recently, however, faricimab, a bispecific antibody that inhibits both VEGF-A and Ang-2, has been approved for use in both neovascular age-related macular degeneration and diabetic macular edema as of January 2022.^53^ Faricimab acts by directly binding and neutralizing both VEGF-A and Ang-2.^54^ Although limited data is available, current findings show the use of the drug in the treatment of DME is comparable to ranibuizumab therapy.^55^

Corticosteroids are another, less effective, medication that can be administered *via* intravitreal injection. These steroids can reduce VEGF expression and have potent anti-inflammatory properties, but the exact mechanism of how they work remains unclear. It is thought that they induce Phospholipase-A2, which regulates the chemotaxis of leukocytes. While corticosteroids are an effective treatment option for inflammatory states in the retina and for treating DME, they pose certain drawbacks as it has been shown they increase the risk of developing cataracts and glaucoma.^56^

As the mainstay procedural intervention, laser pan-retinal photocoagulation (PRP) is a popular option to treat neovascularization. In PRP, focused laser energy is fired into and absorbed by retinal tissue and then converted to thermal energy, destroying the targeted tissue. The subsequent scar tissue that develops in these areas reduces the total amount of ischemic retinal tissue and downstream VEGF production, decreasing aberrant angiogenesis.^57^^,^^58^ There are some inherent risks associated with PRP, however. While this treatment option incurs some inherent damage to the retina, it outweighs the more severe consequences that occur from vessel rupture.^59^ Some adverse effects associated with PRP are retinal detachment, choroidal effusion, and permanent loss of some peripheral and night.^60–63^ PRP-associated retinal detachment and choroidal effusion are exceedingly rare complications, however, and often resolve spontaneously.

### Potential new therapies

Although these therapeutic options have benefited patients with DR, their drawbacks highlight the need for investigating other potential disease mechanisms as a guide for new drug discovery. While many of the current interventional therapies focus on the prevention of angiogenesis, there are few drugs in use aside from faricimab that stabilize the retinal EC-pericyte relationship to prevent pericyte dropout. As stated previously, the angiopoietin-TIE2 system is the primary signaling pathway that determines a stable interaction between ECs and pericytes. Since endogenous Ang-1 from pericytes promotes a stable EC-pericyte relationship, administration of exogenous Ang-1 could be a potential pharmaceutical option.^38^ Alternatively, treatment options that induce pericyte expression of Ang-1 could be investigated to promote an endogenous source. Additionally, inhibitors of Ang-2 expression by pericytes could prevent the destabilization of the Ang-1-TIE2 system and subsequent detachment and migration of pericytes into the intervascular space.^64^ This could be accomplished by the downregulation of the genes responsible for the production of Ang-2.^38^ Of note in streptozotocin-induced diabetic mouse models, the pericyte bridging phenomenon, discussed previously, occurs prior to the reduction in total number of pericytes found in retinal vasculature and was reversible through the exogenous administration of insulin.^47^ Given that this microvascular finding may serve as one of the first definitive signs of the impending downstream pathology seen in DR, treatment at this stage in the disease course may represent a key intervention point during the NPDR phase in order to prevent subsequent irreversible damage to retinal vasculature. Further research is needed to identify additional treatment options that may reverse bridge formation and to ensure these findings can be applied to human retina samples.

Additionally, in studies utilizing mouse models, the role of PDGF in pericyte recruitment was investigated. It was found that signaling involving PDGF-β and PDGF receptor beta (PDGFR-b) is vital in pericyte recruitment at sites of neovascularization.^20^ In conditions of CH, this signaling pathway is negatively affected leading to reduced pericyte recruitment and unstable new vessel growth. Since there are also no current treatment options that promote the recruitment of pericytes to areas of angiogenesis or the re-recruitment to areas of previous dropout, this is a potential therapeutic target for drug development. Although only observed in mouse models, the PDGF-β-PDGFR-b signaling pathway has been shown to be crucial in the proper recruitment of pericytes in the retinal vasculature.^20^ If further research indicates the same pathway is involved in humans, this could be a promising target for treatment through the promotion of pericyte recruitment which in theory could repair a compromised BRB and decrease vascular sensitivity to VEGF.

In addition to pericytes, treatment options centered around the promotion and recruitment of endothelial progenitor cells (EPC) to sites of retinal vasculature damage should also be investigated further. Although not completely understood, preliminary evidence of EPCs and their role in diabetic patients suggest a reduction in number and dysfunction in this cell line for those with varying degrees of CH.^65–67^ EPCs play a complex, yet important role in the repair of damaged vascular tissues, so promoting this cell line early in the disease course could lead to early intervention options during the NPDR phase.^68–70^

## Conclusion

Although great strides have been made in the treatment of patients suffering from DR within the last 15 years, research in the pathogenesis of DR is ongoing and revealing disease mechanisms that once were not fully understood. While further research is still needed, a clear understanding of the healthy state of retinal vasculature along with the disease processes that negatively affect it will provide insight into targets for potential drug therapies at different steps of these pathological pathways. Although current treatments of DR have shown to be effective in ameliorating the disease process of DR, novel drug interventions targeting pericyte dropout could be an adjunct to existing therapies.

## Data Availability

This manuscript was drafted utilizing previously published articles found in the public domain and did not generate any novel data. Therefore, data sharing is not applicable for this manuscript.
